# Familial Monkeypox Virus Infection Involving 2 Young Children

**DOI:** 10.3201/eid2902.221674

**Published:** 2023-02

**Authors:** Pascal Del Giudice, Agnes Fribourg, Laurent Roudiere, Juliette Gillon, Anne Decoppet, Mathieu Reverte

**Affiliations:** Centre Hospitalier Intercommunal de Fréjus-Saint-Raphaël, Fréjus, France (P. Del Giudice, A. Fribourg, L. Roudiere, J. Gillon, M. Reverte);; Agence Régionale de Santé, Toulon, France (A. Decoppet)

**Keywords:** mpox, mpox virus, MPXV, viruses, intrafamilial transmission, parents, young children, infection, infectious diseases, pediatrics, zoonoses, France

## Abstract

We report intrafamilial transmission of monkeypox virus to all members of a family (father, mother, and 2 children). Case reports in young children have been extremely rare during the 2022 mpox outbreak. Their clinical signs were mild, and clinical diagnosis would be difficult without knowledge of the father’s monkeypox virus infection.

Monkeypox virus (MPXV) is a zoonotic orthopox virus. An outbreak of MPXV infections emerged in the spring of 2022 outside Africa, mainly in Europe and the United States, such that on July 23, 2022, the World Health Organization declared the outbreak to be a public health emergency of international concern. During this outbreak, MPXV spread has disproportionately affected gay or bisexual men or other men who have sex with men, which suggests transmission through sexual or intimate contact. However, in August 2022, we observed intrafamilial transmission of the virus to all members of a family (father, mother, and 2 children) in France.

The father, a 30-year-old-man, showed a few papular pustules on his body, including his penis. The pustules began appearing on July 17, 2022. A pustule sample was tested for MPXV and showed a positive PCR result. The wife of the man showed a few pustules on August 2 that were later confirmed to be positive for MPXV by PCR. She had no mucous signs. Both persons were HIV negative.

The couple and their 2 young daughters went on vacation to a campsite in southern France on August 6. Their 4-year-old daughter had a fever (temperature 38°C) and a skin eruption that began on August 5, which consisted of 3 types of lesions: an umbilical pustule (Figure, panel A), papular pustules on an erythematous basis (Figure, panel B), and a disseminated faint erythematous macula (Figure, panels C, D). She also had a bilateral conjunctivitis but no lymphadenopathy or mucosal lesions.

**Figure:**
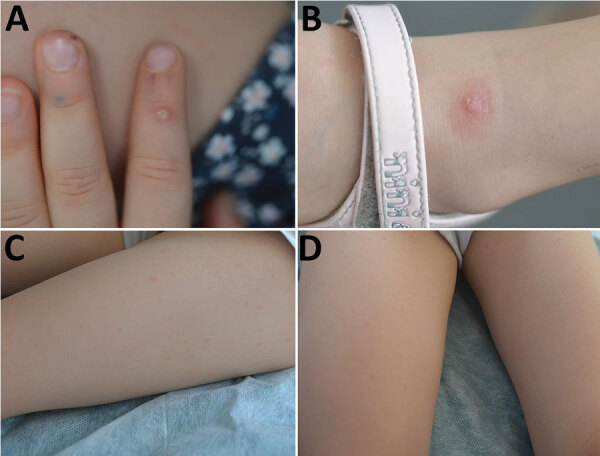
**Figure.** Monkeypox virus lesions for the 4-year old daughter in a family (father, mother, 2 children) infected with the virus, August 6, 2022. A) Umbilical pustule on pulp of the finger; B) papulopustule on the ankle; C, D) faint erythematous rash on the thighs.

On August 9, her 7-year-old sister showed ≈10 asymptomatic micropapular pustules on a discrete erythematous basis (Appendix Figure 1). She had no fever, mucosal lesion, or lymphadenopathy. 

The family was residing at a campsite in southern France, where there is a high burden of *Aedes albopictus* mosquitoes. They were suspected of having either MPXV infection or *Aedes* sp. mosquito bites. A skin scraping from the skin of a micropapular pustule was positive for MPXV by PCR. For all family members, only 1 lesion/person was sampled. Onset symptoms and positive PCR results are shown in the timeline (Appendix Figure 2). The family received isolation instructions and returned home on August 11. The outcome was favorable for all 4 persons. There were no secondary cases at the campsite.

During the ongoing outbreak, cases of MPXV infection in young children have been extremely rare. US data from the Centers for Disease Control and Prevention as of October 7, 2022, reported 26,577 cases in 28 patients (0.1%) <12 years of age (*1*,*2*). Those data were confirmed in Europe. As of August 3, 2022, among 4,663 laboratory-confirmed cases of MPXV infection reported in Spain, only 4 (0.1%) were in children <4 years of age (7, 10, and 13 months, and 3 years) and 12 were in adolescents 13‒17 years of age (*3*). As of August 23, 2022, a total of 41 countries in the World Health Organization European Region have reported 21,098 cases, of which 15 (0.07%) were in persons <15 years of age (*4*).

Intrafamilial transmission to all family members including both children, also appears extremely rare in the 2022 outbreak. We assume that the transmission route was through household direct skin-to-skin contact with their parents.

The clinical signs shown by the children were mild, and clinical diagnosis would be extremely difficult in the absence of knowledge of the MPXV infection of the father. Case reports in young children have been extremely rare in this outbreak (*5*–*7*). Thus, a dermatologic description is lacking in this population. The 2 children in this report had mild general signs. Skin lesions consisted of a few umbilical pustules or papulopustules similar to those reported (*8*–*10*). However, the children also had discrete micropapular pustules on an erythematous basis similar to mosquito bites and a faint erythematous rash. Such skin lesions have rarely been reported in children or adults (*8*–*10*).

Appendix Additional information on familial mpox virus infection involving 2 young children.
