# Supplementary material for: Familial Monkeypox Virus Infection Involving 2 Young Children
Source: Emerg Infect Dis. 2023 Feb;29(2):437–9. doi: 10.3201/eid2902.221674 (PMC9881778; doi:10.3201/eid2902.221674)
Supplement: Appendix — Additional information on familial mpox virus infection involving 2 young children. [file 22-1674-Techapp-s1.pdf]

Article DOI: https://doi.org.10.3201/eid2902.221674

Familial Mpox Virus Infection Involving 2 Young Children

Appendix

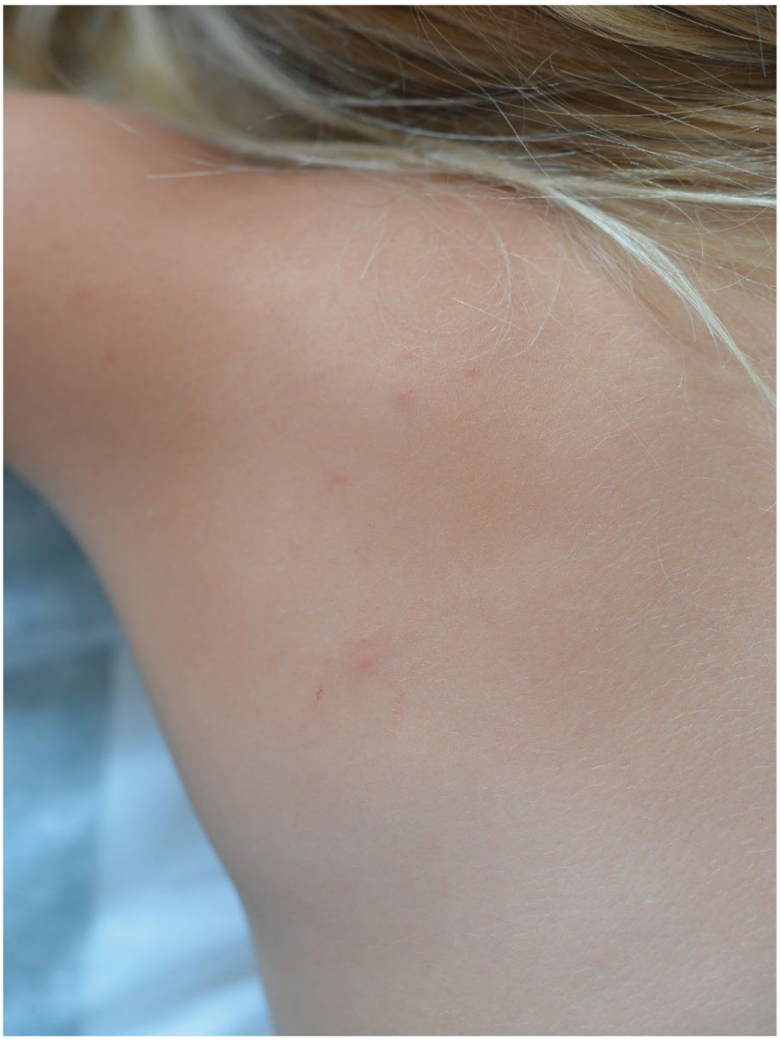

Appendix Figure 1. Micropapular pustule over a discreet erythematous basis similar to a mosquito bite, August 9, 2022.

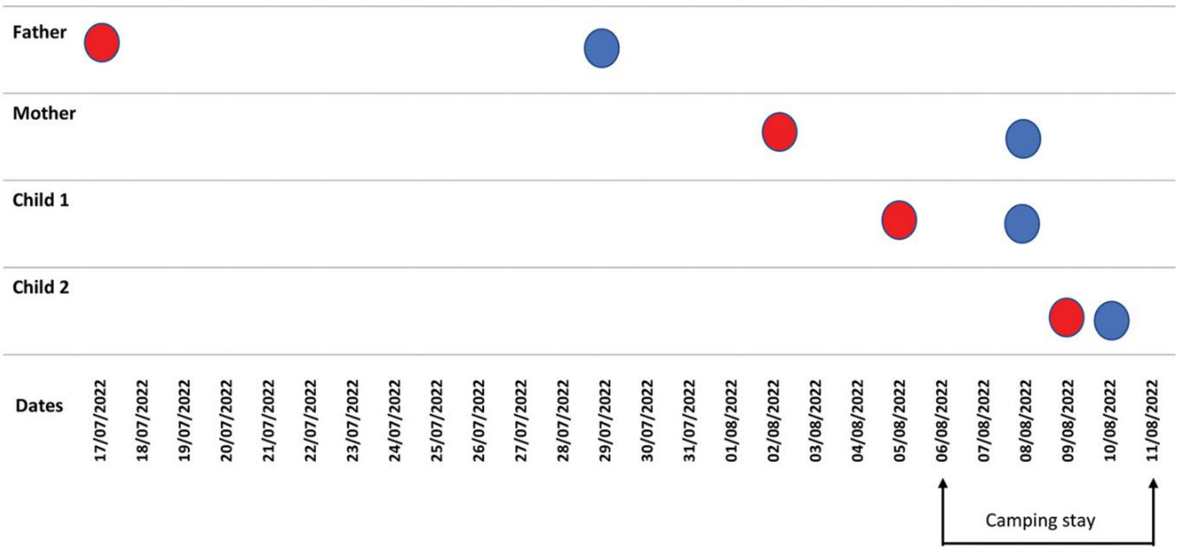

Appendix Figure 2. Timeline of mpox virus infection in each family member. Red dots indicate onset of symptoms, and blue dots indicate sample date and positive PCR result.
